# Pioneers in Care: The Incorporation of Men in Nursing in Spain and Great Britain (1915-1980)

**DOI:** 10.17533/udea.iee.v43n2e10

**Published:** 2025-07-25

**Authors:** Kevin Antonio Jiménez Alcócer

**Affiliations:** 1 Nurse, Master’s degree candidate. Professor and Researcher, Universidad de Costa Rica, Costa Rica, E-mail: alco0525@gmail.com Corresponding author. https://orcid.org/0000-0002-8142-2146 Universidad de Costa Rica Universidad de Costa Rica Costa Rica alco0525@gmail.com

**Keywords:** history, men, nursing, nurses, male, Spain, the United Kingdom, historia, hombres, enfermeros, enfermería, España, Reino Unido., história, homens, enfermeiros, enfermagem, Espanha, Reino Unido.

## Abstract

**Objective.:**

To describe the incorporation of the first male nurses in Spain and Great Britain between 1915 and 1980.

**Methods.:**

This study adopted the analytical-synthetic historical method as epistemological framework to examine the evolution of the appearance of the first male nurses in Spain and Great Britain, supported by documentary sources from databases (SCIELO, SCOPUS, EBSCO, Google Scholar) using descriptors in Spanish, English, and Portuguese. Historical critique was applied at three levels: authenticity, representativeness, and relevance. The analysis combined temporal and thematic perspectives, examining causal factors, such as economic, social, ideological factors.

**Results.:**

In Spain, nursing had segregated roles, women as nurses or midwives and men in the role of interns. In Great Britain, it was exclusively practiced by women. The Spanish Civil War and the dictatorship of Francisco Franco in Spain and the Second World War in Great Britain promoted male incorporation in both countries. The post-war periods and changes in gender roles facilitated a sociocultural transformation, integrating men into nursing and breaking stereotypes. This process redefined the profession, promoting equity and highlighting care beyond gender.

**Conclusion.:**

The historical study reveals that nursing in Spain and Great Britain evolved from an exclusive profession for women toward a more inclusive model, defying stereotypes. Wars and social changes permitted incorporating men, redefining the professional identity. This transformation does not seek to privilege, rather, it seeks to highlight equity and that regardless of the sex or gender care can be provided as long as it has a scientific and ethical basis.

## Resumen

**Objetivo.:**

Describir la incorporación de los primeros hombres enfermeros en España y Gran Bretaña entre 1915 y 1980.

**Metodología:**

Estudio que adoptó el método histórico analítico-sintético como marco epistemológico para examinar la evolución de la aparición de los primeros hombres enfermeros en España y Gran Bretaña, apoyado en fuentes documentales de bases de datos (SCIELO, SCOPUS, EBSCO, Google Académico) utilizando descriptores “historia de la enfermería”, “hombres enfermeros”, “enfermería”, “España” y “Gran Bretaña” en español, inglés y portugués. Se hizo crítica histórica en tres niveles: autenticidad, representatividad y pertinencia. El análisis combinó perspectivas temporales y temáticas, examinando factores causales como son: económicos, sociales e ideológicos.

**Resultados.:**

En España, la enfermería tenía roles segregados: mujeres como enfermeras o matronas y hombres en rol de practicantes. En Gran Bretaña, era exclusivamente ejercida por mujeres. La Guerra Civil española y la dictadura de Francisco Franco en España, y la Segunda Guerra Mundial en Gran Bretaña impulsaron la incorporación masculina en ambos países. Las posguerras y los cambios en los roles de género facilitaron una transformación sociocultural, integrando a los hombres en la enfermería y rompiendo estereotipos. Este proceso redefinió la profesión, promoviendo la equidad y destacando el cuidado más allá del género.

**Conclusión.:**

El estudio histórico revela que la enfermería en España y Gran Bretaña evolucionaron desde una profesión exclusiva de la mujer hacia un modelo más inclusivo, desafiando estereotipos. Guerras y cambios sociales permitieron la incorporación del hombre, redefiniendo la identidad profesional. Esta transformación no buscó privilegiar, sino destacar la equidad y que sin importar el sexo o género se pueda realizar cuidado mientras tenga una base científico-ética.

## Resumo

**Objetivo.:**

Descrever a incorporação dos primeiros enfermeiros na Espanha e na Grã-Bretanha entre 1915 e 1980.

**Métodos:**

Este estudo adotou o método histórico analítico-sintético como referencial epistemológico para examinar a evolução do surgimento dos primeiros enfermeiros na Espanha e na Grã-Bretanha, apoiado por fontes documentais de bases de dados (SCIELO, SCOPUS, EBSCO, Google Acadêmico) utilizando descritores em espanhol, inglês e português. A crítica histórica foi aplicada em três níveis: autenticidade, representatividade e relevância. A análise combinou perspectivas temporais e temáticas, examinando fatores causais como econômicos, sociais e ideológicos.

**Resultados.:**

Na Espanha, a enfermagem tinha papéis segregados: mulheres como enfermeiras ou parteiras e homens como profissionais. Na Grã-Bretanha, era exercida exclusivamente por mulheres. A Guerra Civil Espanhola e a ditadura de Francisco Franco na Espanha e a Segunda Guerra Mundial na Grã-Bretanha promoveram a incorporação de homens em ambos os países. O período pós-guerra e as mudanças nos papéis de gênero facilitaram uma transformação sociocultural, integrando homens à enfermagem e quebrando estereótipos. Esse processo redefiniu a profissão, promovendo a equidade e destacando o cuidado para além do gênero.

**Conclusão.:**

Este estudo histórico revela que a enfermagem na Espanha e na Grã-Bretanha evoluiu de uma profissão exclusivamente feminina para um modelo mais inclusivo, desafiando estereótipos. Guerras e mudanças sociais permitiram a incorporação de homens, redefinindo a identidade profissional. Essa transformação não busca privilegiar, mas sim destacar a equidade e que o cuidado pode ser prestado independentemente de sexo ou gênero, desde que seja baseado em fundamentos científicos e éticos.

## Introduction

Nursing, as social and institutional practice, has maintained a historical link with the female gender role; its transition from informal occupation (based on knowledge transmitted over generations) to formalized profession with scientific status was and continues being a complex process promoted by pioneers like Florence Nightingale. Her work during the War of Crimea (1853-1856) not only revolutionized military health management by applying statistical methodologies and hygiene measures, but also set the epistemological bases of modern nursing through her work “*Notes on Nursing”* during 1859.^1^ Her struggle for disciplinary autonomy, regulated education, and the social recognition of the profession continues influencing on the formation of current and future generations. Due to this, female leadership in nursing is not a mere reflection of ancestral traditions, but the result of a deliberate professionalization process**,** marked by gender, class, and ethnic tensions. Nurses have been agents of global change, from the reform of health systems to the defense of human rights in contexts of crisis. Their impact, although historically invisible, is inseparable from the development of past, present, and future health.^1^

Care, as social activity, has been historically associated with the feminine due to patriarchal structures that linked women with domestic and affective roles, while relegating men to the public and productive sphere. This sexual division of work, rooted since ancient times and reinforced during the Industrial Revolution, was not casual, but a social-order tool that justified the exclusion of women and nursing to subordinated roles.^2^ However, although a minority, male nurses have played a relevant role in the history of nursing since ancient times; their contributions, despite being less documented, have been significant for the development of the profession. Therein, it results key to analyze, from a historical perspective, how men began to undertake active roles within care contexts, which would permit understanding their impact on the construction of an inclusive professional identity and broaden the scope of this discipline.^3^

In this sense, it is essential to investigate the reasons that drove the first male incorporations into nursing, as well as the factors that facilitated their participation.^4^ A paradigmatic case is that of Spain and Great Britain, where - despite language, cultural, and historical differences, the first male nurses emerged during similar periods. This coincidence offers a unique opportunity for a comparative analysis that explores parallelisms and divergence in both contexts.^5^ Hence, the aim of this work was to describe the incorporation of the first male nurses in Spain and Great Britain between 1915 and 1980, highlighting the historical, social, and cultural factors that influenced on their initial insertion. This analysis seeks to enhance comprehension of how gender roles and sociocultural contexts shaped nursing’s transition from occupation to profession, with the participation an actor: male nurses.

## Methods

This study adopted the analytical-synthetic historical method as epistemological framework to examine the evolution of the appearance of the first male nurses in Spain and Great Britain. This dual approach permits decomposing the historical phenomenon into its constitutive elements to then reconstruct it critically, overcoming the limitations of merely chronological analyses. The documentary phase was supported on a database search (SCIELO, SCOPUS, EBSCO Host, Google Scholar) using a trilingual strategy (Spanish, English, Portuguese) with key descriptors, like: history of nursing, male nurses, nursing, Spain and Great Britain, combined with the Boolean operator “AND”. The resulting drafts were subjected to a three-level historical critique process: source authenticity, contextual representativeness, and thematic pertinence. The structural analysis revealed the intrinsic dynamics of the phenomenon through a double approximation: vertical (temporal, establishing meaningful periodization) and horizontal (thematic, examining the economic, social, and ideological dimensions in their interaction). This multi-causal dissection permitted identifying not only the dominant patterns, but specially the latent tensions that configured historical inflection points. The final synthesis transcended the mere sum of evidence that articulated three analysis scales: the particularity of the cases studied, their national context and global influences. This process revealed how structural continuities coexisted with paradigmatic ruptures, offering an understanding of evolution that enriches existing historiography.

## Results

Between 1915 and 1980, an interesting period for analysis took place due to the emergence of the institutionalization of revolutionary or post-war processes in 1915. By 1980, on the other hand, the post-war economic boom ended and the transition to neoliberalism began in countries, like Spain and Great Britain. Both nations underwent transformations that influenced profoundly on diverse aspects of their societies, including the evolution of nursing. These historical changes provided a fundamental framework to comprehend the profession’s development and the insertion of male nurses in both countries.

### From interns to nurses: the first Spanish men in nursing.

In Spain, the first half of the 20^th^ century was deeply influenced by marked political instability that affected social development, as well as educational and professional structures. Although the country adopted a neutrality posture during the First World War (1914-1918), the conflict’s economic and social repercussions had a significant impact in the national territory.^6^ This period was marked by political transformations, such as Primo de Rivera’s dictatorship (1923-1930) and the Second Republic (1931-1936), which promoted initiatives aimed at modernizing the State and endorsing reforms in distinct sectors.^7^ Within the nursing setting, training of professionals was profoundly conditioned by the institutional and sociocultural environment of the time. Nurses and midwives were educated in hospital schools, predominantly run by religious orders, where they received training oriented exclusively to the hospital field. Nurses were specialized in providing direct care to patients, while the midwives performed functions in areas of gynecology and obstetrics, as well as in care during natural births. These professions were strictly reserved for women, in consonance with the social values ​​that associated women with the role of caregivers par excellence.^8^

In contrast, men played the role of interns, an occupation whose formation was carried out in medical schools. This technical preparation included activities, like healing wounds, bandages, vaccinations, and minor surgery. The interns acted principally as medical assistants, both in the public sector and in private consultations, and acquired special relevance in rural zones, where the lack of physicians was a constant.^9^^,^^10^ This professional configuration evidenced a clear sex segregation in the functions and responsibilities assigned. Women, considered culturally suitable for direct care due to their presumed maternal instinct, were trained for care tasks. In turn, men occupied a more technical role, closely related with the medical practice.^8^ It is worth indicating that nursing during this period lacked autonomous professional identity, given that it was deeply subordinated to the medical figure. Physicians were not only in charge of training the nurses, they also regulated their labor practice, evaluated their performance, and defined the standards of the occupation. This dynamic perpetuated a care-based and dependent vision of nursing, limiting its development as a scientific and professional discipline.^11^

The Spanish Civil War and the establishment of the Franco regime marked a turning point in the history of nursing in Spain, causing a significant setback in its professionalization. The Decree of 4 December 1953 unified the studies of nursing, midwives and interns under the figure of the Technical Health Assistant (THA). Nonetheless, despite this formal unification, differentiated training paths and hierarchical work roles persisted. Women continued mostly associated to care tasks in religious hospital environments, while men retained a technical profile, similar to that of the former practitioner/intern. This distinction, under the same professional name, demonstrates the depth of gender barriers in the healthcare field: beyond the title shared, the professional’s sex determined rigidly the type of care provided. This division was reinforced by the context of ideological and educational regression of Francoism, which isolated Spanish nursing from international progress and relegated it to a traditional model, linked to conservative values and immovable gender roles*.*^10^^,^^12^^,^^13^

Nevertheless, after Franco’s death in 1975, Spain experienced a process of opening and transformation in the educational and healthcare sectors; This context favored the evolution of nursing towards a more scientific model and less dependent on its vocational and religious nature.^10^^,^^12^^,^^13^ Between 1977 and 1980, the integration of nursing into the higher education system, through the creation of the University Diploma in Nursing, marked a fundamental milestone. This advance not only permitted the creation of academic programs with a broader and inclusive curricular perspective, but also consolidated the professionalization of nursing, guaranteeing the formation of men and women under equality of conditions and strengthening its autonomy as scientific and professional discipline in the healthcare environment.^8^^,^^10^

### Male nurses from Great Britain (1915 - 1980)

During the course of the 19^th^ century, nursing in Great Britain underwent a profound restructuring in educational terms, accompanied by the consolidation of a marked sexual division of labor.^14^ This phenomenon was rooted in Victorian ideologies, characterized by a separatist conception of the sexes, which attributed strictly differentiated roles to men and women. Within this framework, the nursing profession was practically circumscribed to women, a situation that persisted even in the 20^th^ century.^15^^,^^16^ Contrary to Spain, whose neutrality during the First World War (1914-1918) limited the direct impact on the healthcare setting, Great Britain played an active role in said conflict.^17^^,^^18^ The growing demand for healthcare staff during this period encouraged the massive incorporation of women into nursing. However, the magnitude of the needs also promoted, although incipiently, the participation of men in this occupation, marking the beginning of a paradigm shift in the dynamics of care work.^15^^,^^16^

The interwar period (1919-1939) was an ambivalent period.^19^ On the one hand, the war experience underlined the relevance of nursing and its need to adapt to new demands; on the other hand, the normative and cultural structures reinforced segregation.^20^ Within this context, the enactment of the First Nursing Registration Act in 1919, promoted by the newly created General Nursing Council, established that the nursing practice should be carried out exclusively by women. This legislative act, far from being a mere formalism, consolidated the exclusion of men from training schools, based on logistical and cultural arguments, such as the lack of adequate infrastructure or incompatibility with the social norms of the time.^15^^,^^16^ Nevertheless, in 1922 the first 15 male nurses were officially registered, representing a symbolic disruption in the hegemonic model. In 1923, the election of the first male nurse as a member of the General Nursing Council marked a milestone in the fight for equality in the profession. This representative played a crucial role until 1932, advocating for the inclusion of men as an integral part of the nursing community.^15^^,^^16^

The foundation of the Society of Registered Nurses in 1937 by Edward Glavin constituted substantial progress. This institution was focused on professionalizing men in nursing, fostering their training in modern techniques and addressing the specific challenges of their integration, particularly within the setting of mental health, where the need for qualified personnel was pressing.^15^^,^^16^ Progress towards equality reached an inflection point in 1943, with the merger of the male and female registers into a single general register. This measure, although progressive, generated significant tensions, given that many female nurses perceived this integration as a threat to their position in the profession. The abolition of the Nurses Act in 1949 definitively consolidated the union of both groups, setting the bases for a more-inclusive profession.^15^^,^^16^

Between 1937 and 1945 there was a notable expansion in educational opportunities for male nurses. While at the beginning of this period there were only seven hospitals that accepted them, by its conclusion this figure had increased to 24 transforming institutions. This development evidenced change in the social and professional perception of men in nursing, reducing gaps in access to training.^15^^,^^16^ During the Second World War (1939-1945), the masculine incorporation into nursing increased again due to the emergency generated by the conflict.^21^ Thereafter, the social movements of the 1960s and 1970s challenged gender stereotypes, promoting greater equity in healthcare.^15^^,^^16^

The implementation of the Management Policy by the National Health Service represented another crucial factor in this process. This regulation promoted professional advancement based exclusively on merit and abilities, independent of sex. Due to such, men were able to access leadership positions in nursing, breaking from the tradition that reserved these roles for women.^15^^,^^16^

Lastly, in 1980, the Royal Commission on Health Services established as priority objective to attract men to nursing, promoting their full integration in the profession. This effort culminated with the consolidation of an inclusive model that eliminated the sex barriers mainly, transforming radically the perception and dynamics of nursing in Great Britain.^15^^,^^16^

### Male nurses: a description of their appearance in Spain and Great Britain

The analysis of the incorporation of men in nursing between 1915 and 1980, within the contexts of Spain and Great Britain, permits identifying how, despite cultural and linguistic differences, a transformation emerged in the perception about who could practice this profession. Understanding the historical context that facilitated this change is essential to strengthen the construction of more inclusive and sound professional identity.^22^ In its beginnings, nursing in both countries was profoundly linked with the feminine role, with minimum participation from men in health care.^2^ This minority group dedicated to this task used to be pigeonholed into peripheral roles that, although associated with nursing, were not considered an integral part of the profession. This context marked the start of a transformation process in the nursing professional identity, albeit still far from a definitive consolidation.^23^

War conflicts in Spain and Great Britain were the principal catalyst for this change in both contexts. Wars, as historical events of great impact, tend to transform social perspectives and generate new ways of approaching rising needs, including the offer of health care. During health crises derived from these conflicts, both in the battle fronts and outside of such, the increasing demand for nursing personnel evidenced the need to incorporate more men in this profession.^6^^,^^19^^,^^20^ The massive loss of men during war conflicts and the need for women to assume traditionally masculine roles in industry and other work sectors reconfigured profoundly the sex perceptions within the professional setting, including nursing. This phenomenon, also influenced by the Industrial Revolution and labor reorganization, transformed how professions were understood and assumed during that historical moment.^6^^,^^19^^,^^20^

Simultaneously, nursing began to transcend the hospital confines, acquiring a new professional dimension. This implied a transition from a traditional model of maternal and self-sacrificing care toward a profession that required technical, clinical, and academic training.^24^ This change both broadened the scope of nursing and impacted its structure and language, contributing to the creation of a more inclusive professional gender identity.^25^ Historically, nursing has been marked by being practiced almost exclusively by women, which makes it essential to include gender as a central variable in any study on professional identity. In this sense, it is necessary to more clearly define the framework within which men began their integration into this discipline in both countries.^24^ Gender, understood as a historical and social construction, responds to factors such as the territory, communication, language, and collective and individual representations. The inclusion of men broke with a model that traditionally associated this profession exclusively to feminine sector, enriching and diversifying its professional identity.^25^

Identity, far from being a fixed concept, is a position that subjects adopt regarding their existence and the collectivities surrounding them. This joint identity, influenced by styles, habits, and discourses, positions the individual within a heterogeneous social structure. Therefore, the integration of men in nursing by 1980 was not a fortuitous event, but the result of a series of multifactorial events that culminated in the transformation of the profession.^22^ Finally, the integration of men in nursing and its progressive normalization reflect significant change in the social and professional structures of the time. This process permitted opening the profession to individuals from any sex and gender, enriching its identity and marking progress toward greater equity and inclusion in the health care environment.^22^

## Conclusion

The historical analysis of the male presence in nursing between 1915 and 1980 permits understanding how this profession underwent a paradigmatic transformation in Spain and Great Britain. During this period, nursing stopped being perceived exclusively as a setting reserved for women, paving the way for change in the social and professional structures of both countries. In spite of the cultural and contextual differences, both shared a phenomenon in which the inclusion of men challenged traditional norms tied to gender, configuring a new professional identity.

Construction of a new more inclusive professional identity not only benefits female and male nurses, but also promotes more comprehensive understanding of the profession. History, as a tool to rescue and value the roots of nursing, allows reflecting upon the challenges and progress that marked this change. Particularly, it turns out essential to highlight the impact of incorporating men onto a context in which, for quite some time, they represented a minority in this discipline. This historical perspective honors the path taken and helps those who today have the privilege of being called nurses to comprehend the context that enabled their participation.

It is fundamental to stress that this reflection does not seek to position men above women in nursing, rather, it seeks to reinforce an identity based on equity and on anybody’s capacity, regardless of their gender, to provide care from a scientific and ethical perspective. In this sense, we are called on to delve into the research and analysis of the history of nursing; understanding our past helps us to recognize the current state of the profession and permits our influencing positively on its future, consolidating nursing as a science and essential discipline for the wellbeing of society. 
